# Methylglyoxal Alone or Combined with Light-Emitting Diodes/Complex Electromagnetic Fields Represent an Effective Response to Microbial Chronic Wound Infections

**DOI:** 10.3390/antibiotics14040396

**Published:** 2025-04-10

**Authors:** Firas Diban, Paola Di Fermo, Silvia Di Lodovico, Morena Petrini, Serena Pilato, Antonella Fontana, Morena Pinti, Mara Di Giulio, Emilio Lence, Concepción González-Bello, Luigina Cellini, Simonetta D’Ercole

**Affiliations:** 1 Department of Pharmacy, University “G. d’Annunzio” Chieti-Pescara, Via dei Vestini 31, 66100 Chieti, Italy; firas.diban@unich.it (F.D.); silvia.dilodovico@unich.it (S.D.L.); serena.pilato@unich.it (S.P.); antonella.fontana@unich.it (A.F.); morena.pinti@phd.unich.it (M.P.); mdigiulio@unich.it (M.D.G.); l.cellini@unich.it (L.C.); 2 Department of Medical, Oral and Biotechnological Sciences, University “G. d’Annunzio” Chieti-Pescara, Via dei Vestini 31, 66100 Chieti, Italy; paola.difermo@unich.it (P.D.F.); morena.petrini@unich.it (M.P.); 3 Centro Singular de Investigación en Química Biolóxica e Materiais Moleculares (CiQUS), Departamento de Química Orgánica, Universidade de Santiago de Compostela, Jenaro de la Fuente s/n, 15782 Santiago de Compostela, Spain; emiliojose.lence@usc.es (E.L.); concepcion.gonzalez.bello@usc.es (C.G.-B.)

**Keywords:** Methylglyoxal, light-emitting diodes, complex magnetic fields, microbial infection, chronic wound

## Abstract

**Background:** antimicrobial resistance represents a critical issue leading to delayed wound healing; hence, it is necessary to develop novel strategies to address this phenomenon. **Objectives:** this study aimed to explore the antimicrobial/anti-virulence action of Methylglyoxal-MGO alone or combined with novel technologies such as Light-Emitting Diodes-LED and Complex Magnetic Fields-CMFs against resistant clinical strains isolated from chronic wounds. **Methods:** characterized planktonic *Staphylococcus aureus*, *Pseudomonas aeruginosa*, and *Candida albicans* isolates were used. Antimicrobial activity was evaluated by measuring optical density, Colony Forming Units-CFU, and synergy between MGO/LED or CMFs. Cellular membrane permeability by propidium iodide fluorescence and fluidity by Laurdan generalized polarization measurements were performed. *P. aeruginosa* motility was tested using the soft agar method. A docking study was performed to evaluate the possible interaction between MGO and urease in *P. aeruginosa*. **Results:** single/combined treatments showed significant antimicrobial activity. Major CFU reduction was detected after CMFs/MGO+CMFs application on *C. albicans*. Treatments exhibited significant changes in membrane permeability and fluidity. The treatments decreased *P. aeruginosa* motility with a major reduction after LED application. Docking analysis showed that MGO could bind with *P. aeruginosa* urease leading to defective folding and functional alterations. **Conclusions:** the results suggest that these treatments could represent promising and green therapeutic solutions against resistant isolates from chronic wounds.

## 1. Introduction

Antimicrobial resistance (AMR) represents a serious challenge to public health. This phenomenon emerged gradually after the discovery of antibiotics due to their misuse, provoking microorganisms to develop new methods to withstand the treatment [1]. Traditional antibiotics affect microbial cells by interfering with cell wall synthesis, membrane permeability disruption, protein production, and DNA/RNA synthesis. On the other hand, some microorganisms have developed resistance toward multiple classes of antibiotic compounds and showed increased pathogenicity including ESKAPE bacteria (*Enterococcus faecium*, methicillin-resistant *Staphylococcus aureus*, *Klebsiella pneumoniae*, *Acinetobacter baumannii*, *Pseudomonas aeruginosa*, and *Enterobacter* species). In particular, chronic infections, including wound infections, represent a critical issue in which AMR plays a key role. Among these microorganisms, *Staphylococcus aureus* and *Pseudomonas aeruginosa* represent the two most commonly co-isolated microorganisms from chronic wound ulcers, and their co-existence is linked to increased virulence and poor treatment outcomes [2,3]. Also, recurrent microbial infections are considered a primary cause of chronic inflammation contributing to persistent and delayed wound healing [4].

*S. aureus* acts as pioneer for the attachment of *P. aeruginosa* that, in turn, promotes an invasive phenotype of *S. aureus*. In the first step of colonization, their relationship is competitive but over time a shift occurs, leading to a synergistic interaction between the two species and this interaction results in increased tolerance to traditional treatments for chronic wound infections [5]. The presence of *C. albicans*, in polymicrobial inter-kingdom yeast–bacterial biofilms in chronic wounds, increases the tolerance of *S. aureus* to antibiotics. The interaction between *C. albicans* and *S. aureus* also leads to increased virulence, resulting in increased toxin production, making the infection more dangerous [6]. Additionally, the interactions between *C. albicans* and *P. aeruginosa* are more complex than those with *Staphylococcus*. At the same time, antagonistic and synergistic interactions are observed. For example, Bandara et al. demonstrated that *P. aeruginosa* quorum induces fluconazole resistance in *C. albicans* through upregulation of efflux pumps and ergosterol biosynthesis [7].

AMR phenomenon highlighted the importance of involving new strategies to manage these complex infections, including enhanced wound care, targeted antimicrobial therapy, and biofilm disruption.

Among multiple approaches, Light-Emitting Diode (LED) and Complex Electromagnetic Fields (CMFs) are considered effective and safe methods for antimicrobial applications. LED therapy is widely used in healthcare, and the positive effects observed in wound therapy include a reduction in infectious and inflammatory processes, edema, and inflammatory cell counts, along with stimulation of microcirculation and promotion of new blood vessel formation [8,9,10]. In particular, LED therapy is used to treat fungal and bacterial infections caused by several pathogens [11,12,13] and red light therapy demonstrated high tolerance (up to 320 Joules/cm^2^) and low adverse effects after application [14]. On the other hand, antimicrobial treatment using Complex Electromagnetic Fields (CMFs) is another emerging approach to treat the infections associated with resistant microorganisms. In the literature, magnetic field studies detail multiple antibacterial activities on the growth, cell morphology, antibiotic sensitivity, and biofilm formation [15,16,17]. CMFs exert antifungal and anti-virulence action, in particular against a clinical resistant *C. albicans* strain in terms of biofilm reduction and alteration of the lipidic structure of the membrane without any cytotoxic effects on gingival fibroblasts [16], together with complete wound closure at 48 h and increased collagen production at 7 days [17]. Electromagnetic fields have also demonstrated the ability to promote osteogenesis and to substantially improve the wound healing process [18,19]. The majority of studies primarily focus on investigating the impact of environmental factors on human health, in particular the exposure to extremely low-frequency fields generated by devices operating at a 50 Hz frequency. The safety of electromagnetic fields (100 μT and 500 μT) during long-term exposure was elaborated by Akdag et al. with no significant effect on apoptotic processes, oxidative damage, and reproductive characteristics [20].

Regarding wound treatment, Manuka honey has been used as a topical medication based on demonstrated antimicrobial activity [21,22]. This is primarily attributed to the presence of the reactive Methylglyoxal (MGO), formed during the early stage of Maillard reaction and through the degradation of carbohydrates in foods and beverages during processing, cooking, and prolonged storage [23,24].

Several authors reported the antimicrobial properties of MGO regarding anti-urease activity against *Helicobacter pylori*, inhibition of *E. coli* and *S. aureus* growth, and *P. aeruginosa* biofilm formation [25,26,27]. Lu et al. reported several changes in the cell morphology after the application of MGO-rich Manuka honey against Gram-positive/negative bacterial strains. In particular, the cell length was different for all strains and the DNA was condensed in Gram-positive bacteria [28]. Related to the mechanism of action, it has been suggested: (i) multiple effects on gene expression [29], (ii) chemical modification of proteins involved in the bacterial cell membrane that alters membrane permeability, as well as cell energy metabolism [30], (iii) opening of calcium ion channels resulting in the alteration of the influx of calcium ions into the cell [31], (iv) inhibition of urease enzyme, a virulence factor found in several pathogenic bacteria, which is essential in the colonization of the host organism and in maintenance of bacterial cells in tissues [25,26,32]. Moreover, studies suggest that MGO can enhance the antibacterial activity of other compounds, including synthetic antimicrobials, potentially reducing the required dosage of conventional antibiotics, and resistance development [33,34]. For example, MGO showed synergistic antimicrobial activity with compounds such as linezolid against *Staphylococcus aureus* ATCC 29213 [33], and chitosan against *Escherichia coli* ATCC 8739 and *Pseudomonas aeruginosa* ATCC 9027 tackling the antibiotic resistance phenomenon [34].

Therapy combination, which involves the simultaneous use of two or more antimicrobial agents to treat infections, has been promoted as a strategy for reducing the emergence of antimicrobial-resistant strains. In order to identify novel effective eco-sustainable therapies, the aim of this study was to evaluate the antimicrobial/anti-virulence actions of MGO, LED, and CMFs alone and MGO combined with LED and CMFs as follows:

(i) To investigate the inhibitory effect of LED and CMFs therapy, alone and combined with MGO against clinical pathogenic isolates of *S. aureus*, *P. aeruginosa*, and *C. albicans*;
(ii) To evaluate the effect of LED, CMFs, and their synergistic combination with MGO on *P. aeruginosa* swimming, swarming, and twitching motility;
(iii) To assess the potential changes in cellular membrane permeability and fluidity induced by LED, CMFs, and their synergistic combination with MGO on *S. aureus*, *P. aeruginosa* and *C. albicans* strains;
(iv) To determine the potential interaction between MGO and a target enzyme of *P. aeruginosa* by docking analysis.

Through the investigation of these combined therapeutic approaches, this study aims to contribute to the development of novel, non-antibiotic strategies for combating multi-drug resistant MDR pathogens and enhancing the management of chronic wounds.

## 2. Results

### 2.1. Antimicrobial Activity of MGO

The antimicrobial effect of MGO was evaluated against resistant clinical isolates of chronic wound infections in terms of MIC and MBC/MFC determination. The bacterial strains were multidrug resistant strains and expressed positive resistance genes (while *C. albicans* X3 was sensitive to antifungals) and all microbial strains showed high ability to form biofilm (Supplementary Materials Tables S1 and S2). As shown in Table 1, the MIC values for MGO ranged from 64 µg/mL to 128 µg/mL for *S. aureus* strains, 256 µg/mL for *P. aeruginosa* strains, and 4096 µg/mL for *C. albicans* X3. The MBC/MFC values were always like the MIC values.

### 2.2. Planktonic Optical Density

**Figure 1:**
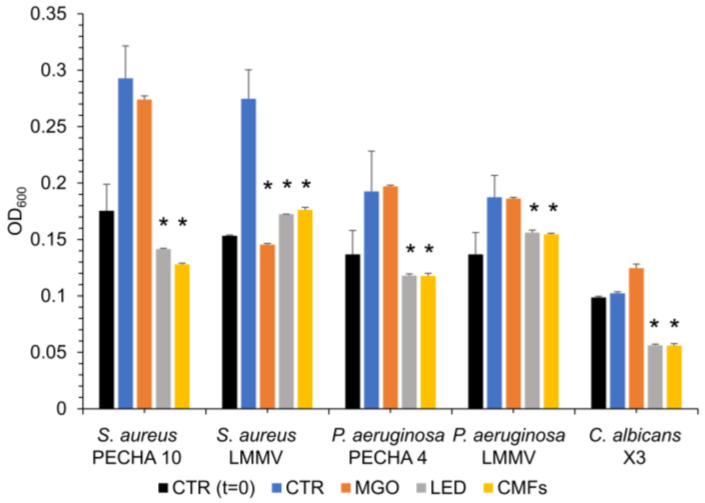
Optical density at 600 nm (OD_600_) values of the strains treated with MIC values of MGO (22 min), LED (17 min), and CMFs (22 min). * Statistically significant values compared to the control (CTR, blue bar) (*p* < 0.05).

The effect of MGO and/or novel technologies was analyzed by optical density observation. Figure 1 shows the planktonic OD_600_ values of *S. aureus*, *P. aeruginosa*, *C. albicans*, clinical strains, in the presence of MIC values of MGO (22 min), LED application (17 min), and CMFs application (22 min). Statistically significant (*p* < 0.05) OD_600_ reduction was recorded in the presence of LED and CMFs, with respect to the control (the OD_600_ of non-treated microbial broth culture, after the same incubation time of the treated wells, 22 min). MGO MIC values, after 22 min of treatment, affected significantly (*p* < 0.05) only the *S. aureus* LMMV OD_600_ value.

### 2.3. Synergism

The combined effect of MGO and/or novel technologies were analyzed. LED and CMFs were able to decrease the total number (OD_600_ value) of microbial cells immediately after the treatment. Then, they were tested to detect their synergism with MGO. The best synergistic combinations of LED/CMFs and MGO are shown in Table 2 with the values of the Fractional Inhibitory Concentration Index (FIC I) for each strain tested. Synergisms (FIC I ≤ 0.5) were recorded for all strains. For *S. aureus* LMMV, *P. aeruginosa* PECHA 4 and *C. albicans* X3 with different synergistic concentrations of MGO were obtained when treated with LED and CMFs. For *C. albicans* X3, the combination between MGO and LED/CMFs reduced the MIC value from 4096 µg/mL to 16 µg/mL and 4 µg/mL, respectively (eight and ten dilution steps lower than the MIC value). Antagonism (FIC I ≥ 4.0) or additive (FIC I > 0.5–4.0) effect was not detected.

### 2.4. CFU/mL Reduction

**Figure 2:**
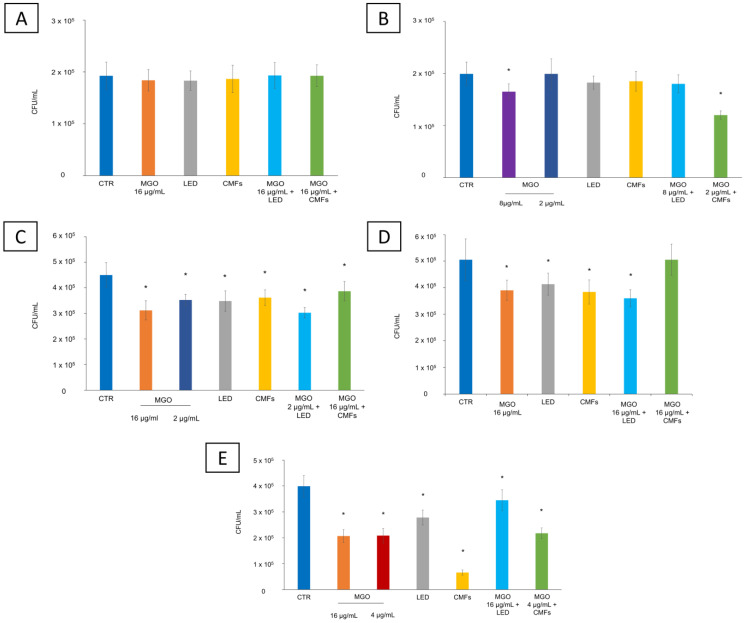
Antimicrobial effect of different treatments on the tested strains. CFU/mL planktonic count of (**A**) *S. aureus* PECHA 10, (**B**) *S. aureus* LMMV, (**C**) *P. aeruginosa* PECHA 4, (**D**) *P. aeruginosa* LMMV, (**E**) *C. albicans* X3, treated with MGO at synergistic concentrations, LED and CMFs, alone and combined with MGO at synergistic concentrations along with non-treated sample (control-CTR). * Statistically significant values compared to the control (*p* < 0.05).

The effect of the combination of MGO and/or novel technologies was analyzed in terms of CFU/mL reduction. Figure 2 shows the antimicrobial effect of different treatments on the selected strains in terms of CFU/mL. *S. aureus* PECHA 10 and LMMV, *P. aeruginosa* PECHA 4 and LMMV, and *C. albicans* X3 were treated with all selected synergistic concentrations of MGO obtained with LED and CMFs.

Regarding the reduction in the *S. aureus* viable count, only *S. aureus* LMMV treated with 8 µg/mL MGO alone and MGO (2 µg/mL) + CMFs showed a significant reduction (*p* < 0.05) of 17.1% and 39.7%, respectively. The other conditions were not effective against *S. aureus* strains (Figure 2A,B).

The treatment with MGO (at the synergistic concentrations of 16 µg/mL, 4 µg/mL, and 2 µg/mL), LED, and CMFs significantly reduced the microbial viable count of *P. aeruginosa* and *C. albicans* strains with respect to the control. In particular, the counts of *P. aeruginosa* strains were significantly (*p* < 0.05) reduced by each treatment: for *P. aeruginosa* PECHA 4, MGO 16 µg/mL caused a reduction of 30.7%, MGO 2 µg/mL of 21.7%, LED of 22.6%, CMFs of 19.6%, MGO (2 µg/mL) + LED of 32.8%, MGO (16 µg/mL) + CMFs of 14.1% (Figure 2C). For *P. aeruginosa* LMMV, MGO 16 µg/mL caused a reduction of 22.8%, LED of 18.2%, CMFs of 24.03%, and MGO (16 µg/mL) + LED of 28.8%. MGO (16 µg/mL) + CMFs did not reduce *P. aeruginosa* LMMV counts (Figure 2D). CMFs significantly reduced the count of *C. albicans* X3 by 83.5% (Figure 2E). Furthermore, treatment with MGO (both at the synergistic concentration of 16 µg/mL and 4 µg/mL) caused a significant reduction of 47.8% and 48.2%, respectively, and the MGO (4 µg/mL) + CMFs of 45.5%. LED promoted a significant reduction of the *C. albicans* X3 CFU/mL of 30.3% and the MGO (16 µg/mL) + LED of 13.6% (*p* < 0.05).

### 2.5. Membrane Permeability

**Figure 3:**
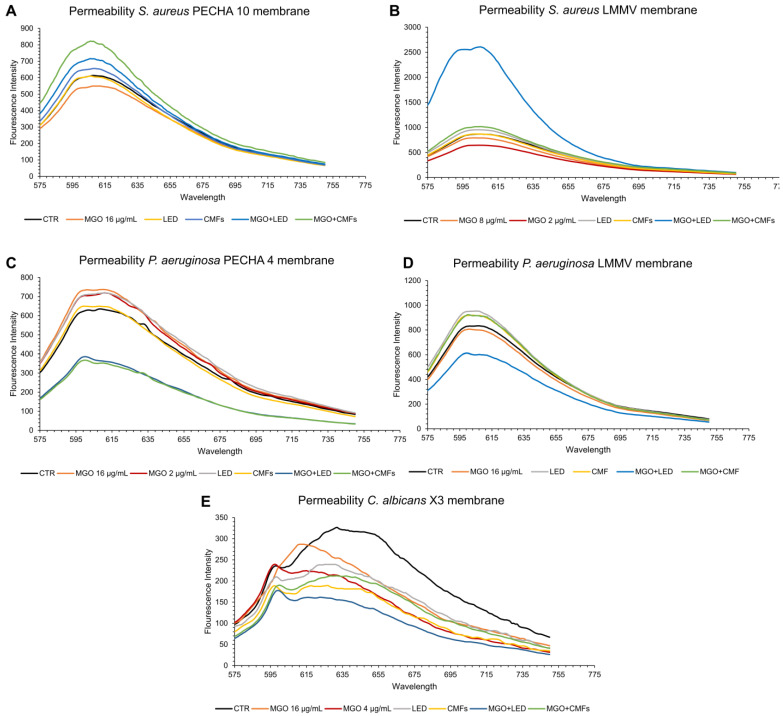
Membrane permeability changes in presence of MGO (at synergistic concentration), LED, CMFs, best synergistic combination MGO + LED, and MGO+CMFs of (**A**) *S. aureus* PECHA 10, (**B**) *S. aureus* LMMV, (**C**) *P. aeruginosa* PECHA 4, (**D**) *P. aeruginosa* LMMV, (**E**) *C. albicans* X3.

The permeability test aimed to detect this activity by evaluating the permeability changes after each single/combined treatment of MGO and the tested technologies. The membrane permeability changes in all the tested strains were determined by propidium iodide (PI) fluorescence intensity. The change in membrane permeability could provide a better understanding of the mechanism of action of MGO alone or combined with LED/CMFs. Figure 3 shows the results obtained with MGO and novel strategies, alone and in combination.

Regarding the bacterial cells, an increase in the fluorescence intensity was observed in the *P. aeruginosa* strains treated with LED, in *S. aureus* PECHA 10 treated with MGO (16 µg/mL) + LED, and *S. aureus* LMMV treated with MGO (8 µg/mL) + LED, indicating an increase in the membrane permeability of the bacteria. Likewise, when the *S. aureus* strains and *P. aeruginosa* LMMV were treated with the MGO+CMFs (synergistic concentrations of MGO were 16 µg/mL for both *S. aureus* PECHA 10 and *P. aeruginosa* LMMV, and 2 µg/mL for *S. aureus* LMMV) the bacterial membrane became permeable and PI fluorescence increased.

Regarding the fungal cells, MGO, LED, and CMFs, including their synergistic combinations with MGO, did not change the *C. albicans* X3 membrane permeability.

### 2.6. Membrane Fluidity

Fluidity test aimed to detect this activity by evaluating the fluidity changes after each single/combined treatment of MGO and the tested technologies. The changes in membrane fluidity caused by different treatments were evaluated by GPexc values. Higher Laurdan GPexc values correspond to a reduction in membrane fluidity and correlate to stress on the geometric packing of the membrane phospholipids. As shown in Table 3, GPexc values showed significant differences compared to the control, which were detected when the microorganisms were treated with MGO, LED, and CMFs alone, except for *P. aeruginosa* PECHA 4 and *C. albicans* X3 treated with CMFs. In particular, the treatments were able to reduce the membrane fluidity of *S. aureus* LMMV and *P. aeruginosa* strains and increase in *S. aureus* PECHA 10.

MGO+CMFs and MGO+LED modified the *P. aeruginosa* PECHA 4 and *C. albicans* X3 membrane fluidity.

### 2.7. Swimming, Swarming, and Twitching Motility

**Figure 4:**
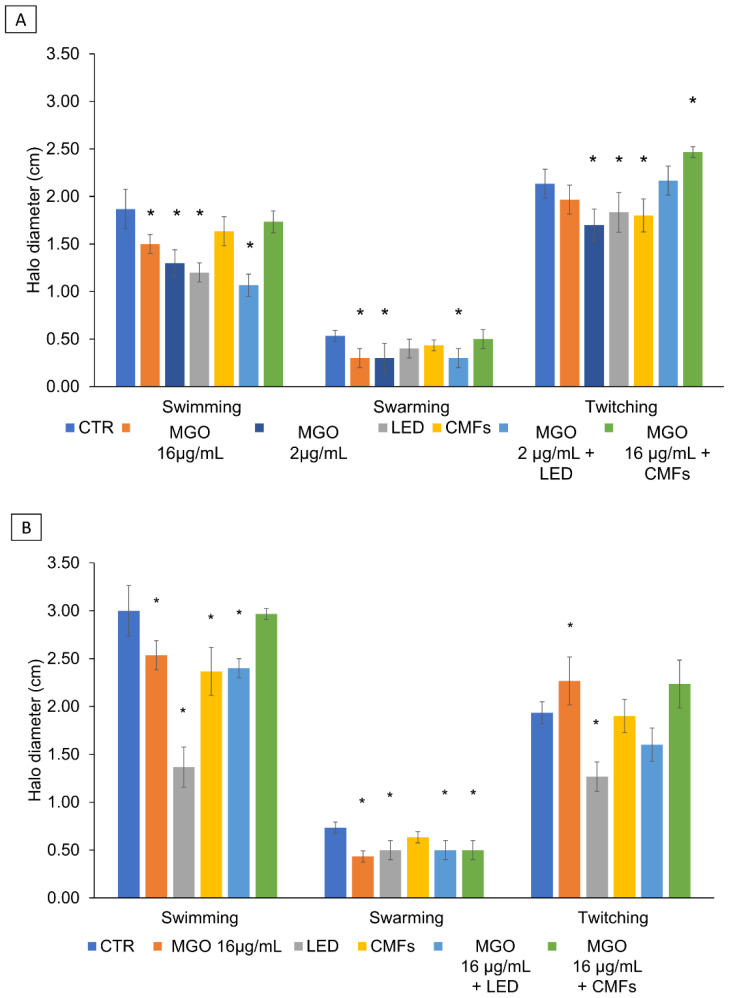
Effect of MGO, LED, CMFs, best synergistic concentrations MGO+LED, and MGO+CMFs on the *P. aeruginosa* PECHA 4 (**A**) and *P. aeruginosa* LMMV (**B**) motility. * Statistically significant values compared to the control (*p* < 0.05).

The action of MGO and/or novel technologies on motility were determined. The soft agar motility assay was performed to evaluate the effect of MGO, LED, CMFs, and their synergistic combinations on *P. aeruginosa* swimming, swarming, and twitching motility. As shown in Figure 4A,B, for *P. aeruginosa* strains, a significant swimming and swarming halo reduction was detected in the presence of MGO (16 µg/mL and 2 µg/mL) alone and combined with LED, with respect to the control (*p* < 0.05). LED significantly reduced the *P. aeruginosa* PECHA 4 swimming and twitching motility (Figure 4A), and the *P. aeruginosa* LMMV swimming, swarming, and twitching motility (Figure 4B), with respect to the control (*p* < 0.05). A significant twitching *P. aeruginosa* PECHA 4 and swimming *P. aeruginosa* LMMV halo reduction was detected in the presence of CMFs (*p* < 0.05). MGO (16 µg/mL) + CMFs significantly affected the *P. aeruginosa* LMMV swarming motility with respect to the control (*p* < 0.05). The intra-group evaluation showed that the (MGO + LED) caused a significant reduction in the swimming motility of *P. aeruginosa* PECHA 4 compared to MGO alone. On the contrary, LED alone caused a significant reduction in the swimming motility of *P. aeruginosa* LMMV compared to MGO and the (MGO + LED). Also, each single treatment reduced the swimming motility of both strains in comparison to the (MGO + CMFs). Finally, (MGO + LED) and (MGO + CMFs) increased the twitching motility of *P. aeruginosa* PECHA 4 compared to the results of single treatments.

### 2.8. Docking Studies

**Figure 5:**
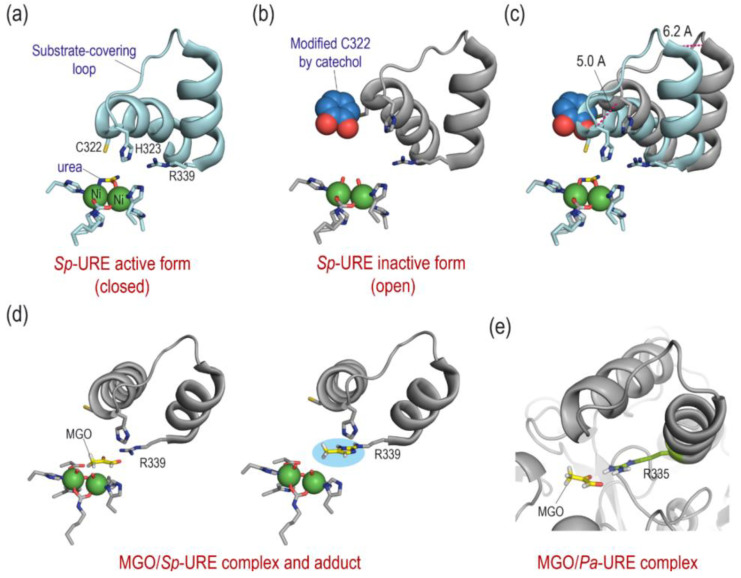
(**a**,**b**) Arrangement of the substrate-covering loop observed in the crystal structures of the Sp-URE enzyme complexed with urea (PDB ID 6QDY, (**a**) and inactivated by catechol (PDB ID 5G4H, (**b**). (**c**) Superposition of (**a**,**b**) highlighting the more open conformation of the inactive form of the enzyme. The distance differences between both loop arrangements are shown as dashed lines. (**d**,**e**) Proposed binding mode of MGO in the active site of Sp-URE (**d**) and Pa-URE (**e**) enzymes obtained by molecular docking and the corresponding Sp-URE adduct (2-aminoimidazole derivative) through the guanidinium group in R339. Relevant side chain residues are shown and labeled. Catechol-modified residue C322 and Ni(II) ions are shown as spheres.

These studies carried out aimed to investigate if the binding between MGO and *P. aeruginosa* urease (Pa-URE) can be a possible mechanism of action. Docking studies using the GOLD program version 2021.3.0 and the enzyme coordinates observed in the crystal structure of the *Sporosarcina pasteurii* urease (Sp-URE) enzyme inactivated by catechol (PDB ID 5G4H) were performed to explore the potential capacity of MGO to cause the covalent modification of the conserved residue R339. The binding of MGO against the *P. aeruginosa* urease (Pa-URE) enzyme was also studied using a homology model created using AlphaFold method. The results revealed that MGO would bind close to the guanidinium group in residues R339 and R335 (conserved) and in a suitable arrangement to trigger the chemical modification of these residues (Figure 5).

## 3. Discussion

The microbial colonization of the wound is responsible for the delay in the healing process and, therefore, the chronicity of the infection [35]. The treatment of wound pathogens is considered a challenge that requires research into new eco-sustainable strategies to tackle microbial proliferation. Recent studies focus on constructing medical devices with immunomodulatory function in order to manage inflammation, along with the antimicrobial action against the resistant bacteria within the chronic wound site [36,37]. The present work aimed to observe the influence of a natural compound, Methylglyoxal (MGO), alone and in combination with novel technologies such as LED and CMFs on planktonic multidrug resistant clinical strains isolated from chronic wounds.

The results of this study confirmed the inhibitory concentrations of MGO (MIC, MBC) reported by other studies for *P. aeruginosa* and *S. aureus* [27,38]. Manuka honey was used as a treatment for wound healing due to its active components (Methylglyoxal, hydrogen peroxide, sugars, phenolic compounds, and bee defensin-1). The activity of isolated components, especially MGO, was screened against various microbial species. It was also demonstrated that MGO leads the non-oxidative antimicrobial activity (in the absence of hydrogen peroxide) and it showed significant growth reduction against *B. subtilis*, *E. coli*, and *S. aureus* after treatment with 1–4% of Manuka honey [28]. Moreover, MGO showed antibacterial and antibiofilm effects against *S. aureus* and *P. aeruginosa* in free-living and sessile forms but with concentrations greater than this study [27,39]. MGO (4%) in nanocomposite of bacterial cellulose showed activity against the most common wound pathogens including antibiotic-resistant *S. aureus* [40].

The results obtained after observation of the OD_600_ reduction in the different broth cultures after all single treatment applications was encouraging to proceed with the study of viable cell count. The results of this study showed that LED and CMFs alone caused a reduction in the viable microbial count of the tested microorganisms in different percentages with a significant effect against *P. aeruginosa* strains and *C. albicans*. Several studies reported similar results after LED application but with different wavelengths, time of irradiation, with photosensitizer or without [41,42,43]. When MGO was combined with LED and CMFs, a relevant antimicrobial effect was detected and this emphasized the importance of these combinations.

In addition, in this study, there was a significant reduction in the viable count when LED and MGO were combined suggesting a synergistic action. No tests were reported in the literature regarding this combination, but Orlandi et al. showed that the blue light and forest honeydew honey had greater killing action against *P. aeruginosa* than single treatment [44].

The results of this study demonstrated a significant reduction of 39.7% of the viable count of *S. aureus* LMMV after the application of MGO and CMFs, for 22 min at a frequency ranging from 6 to 70 Hz, supporting the useful antibacterial activity of the complex magnetic fields. The outcomes of the test depended on several factors including magnetic flux density, frequency, and duration. Magnetic fields with higher intensity and frequency showed antibacterial activity against *E. coli* and *S. aureus* and caused a reduction in the cell count for both bacteria [45,46]. Inhan-Garip et al. reported a morphological alteration in *S. aureus* related to cytoplasmic changes, upon exposure to ELF-EMF (50 Hz at 0.5 mT) but for 120 min application time [47]. In this study, 22 min of exposure to CMFs was able to induce a higher reduction in the viable count of *C. albicans*, both alone (83.5% reduction) and in combination with MGO (48.2% reduction). In a previous study, D’Ercole et al. showed the efficacy of CMFs against *C. albicans* reporting a reduction in cell viability, hyphal morphogenesis, adhesion, and biofilm formation on titanium disks [16]. The results obtained with CMFs were in agreement with Novák et al., which demonstrated a significant decrease in CFU numbers of the yeast *Saccharomyces cerevisae* after 20 min of exposure to a low-frequency magnetic field [48]. It is important to notice that CMFs affected the studied strains in a strain-dependent manner in accordance with the results of Fojt et al. [49]. CMFs significantly reduced the *C. albicans* CFU/mL inducing abnormal sphingolipid–ergosterol domains resulting in reduced microbial growth [17] and when they were combined with MGO, no statistical differences were obtained with respect to MGO alone. MGO acts on fungi nuclear division inhibition by vacuolar morphology alterations and probably a variation in the membrane lipids can reduce the efficacy of MGO [50].

It is important to mention that the parameters of CMFs set in this study (frequency ranging from 6 to 70 Hz, magnetic induction of 6–95 µT, and exposure time of 22 min) can be considered a safe method as detailed by Zanotti et al. [51].

In the literature, MGO was studied as an antimicrobial agent against several microbes and it demonstrated a significant reduction in bacterial growth as mentioned in Rabie et al. [52]. The ultrastructural study showed a change in the morphology of the bacterial membrane in which the bacteria were rounded with membrane damage. This change, in addition to other suggested mechanisms of action of MGO, was encouraging to measure cellular determinants that express any change in the composition or the integrity of the cellular membrane. Thus, this study aimed to detect a possible mechanism of action by evaluating the permeability and fluidity changes after each single/combined treatment of MGO and the tested technologies. In detail, an increase was observed in the membrane permeability of the bacterial strains treated with LED, alone and combined with MGO, and with CMFs combined with MGO. Instead, the treatment decreased the fluorescence of *C. albicans* samples under LED and CMF application suggesting no influence on membrane permeability. The controversial results between the CFU reduction and permeability changes in the tested microbial strains could be attributed to antimicrobial action on the cells but not related to the membrane integrity as found in *C. albicans* results (for example, high CFU reduction with decreased PI fluorescence). On the contrary, Sztafrowski et al. showed that alternating the magnetic field (50 Hz, 35 mT) increased the permeabilization of *C. albicans* cells with growth reduction, probably because of the longer duration of irradiation (24 h) [53].

On the other hand, fluidity measurements showed that each treatment changed the membrane fluidity of both bacteria and *C. albicans*, independently of the strain. Results obtained after irradiating *C. albicans* with CMFs are in accordance with previous results of Di Lodovico et al. [17]. In this study, CMFs alone and in combination with MGO reduced the membrane fluidity, indicating the switch to the liquid order phase and high content of saturated membrane phospholipids and, consequently, increased rigidity [17]. It was demonstrated that treatment with ELF-EMF (50 Hz, 1 mT for 2 h) alters the membrane potential of both Gram-positive and Gram-negative bacteria, affecting specific pathways associated with an increase in pH or ionic fluxes, resulting in membrane hyperpolarization [54]. The results of the fluidity study expressed controversial changes in the GPexc values for each treatment which could be difficult to translate into a direct consequence on the microbial cell growth or functionality. Nevertheless, the change in fluidity and membrane fatty acid composition remains an important factor that could affect either the microbial adhesion [55] on the surfaces or the resistance to other treatments [56,57].

*P. aeruginosa* motility plays a crucial role as a virulence factor promoting several activities that contribute to the pathogenicity and biofilm formation. Generally, the surface motility can be flagellar-mediated swimming motility, collective group swarming motility, or type IV pili-mediated surface twitching [58]. In this study, there was a reduction in the *P. aeruginosa* swimming and swarming motility after MGO application affecting the movement and the ability to cause systemic colonization. Rabie et al. showed that MGO affected the motility of *P. aeruginosa* and this reduction could be attributed to the reduced number of flagella and fimbriae existing on the bacterial cell [52]. Similarly, in this study, LED application reduced the *P. aeruginosa* strain’s motility; this result was supported by Tuttobene et al. in the study about blue light (462 nm) [59]. LED was able to reduce twitching but the cultural analysis is not enough to justify the effect. The lack of the flagellar or *pilT* gene expression represents a limit of this study and further molecular analysis will be carried out to better explain this different effect on the two strains. CMFs reduce *P. aeruginosa* twitching and swimming motility and it is considered important in controlling the bacterium adhesion capability and biofilm formation [60]. The combination of CMFs and MGO significantly reduces the swarming motility of *P. aeruginosa* LMMV. On the contrary, Raouia et al. [61] revealed an increase in *P. aeruginosa* swarming and swimming motility when they used static magnetic fields—SMFs with parameters different from this study (6 h of exposure to SMFs at 200 mT).

Several mechanisms of action for MGO have been proposed and the compound probably has several effects on the bacteria. The docking studies carried out aimed to provide knowledge on one of these possibilities, which could be quite plausible considering the reactivity of the compound, as well as the structural characteristics of the target. This study aims to contribute to analyzing the reason for this possible interaction from both a structural and chemical point of view. Among the several options suggested [30], the urease (URE) enzyme inhibition was considered for the intrinsic strong reactivity of MGO against nucleophilic species, such as arginine, lysine, and cysteine residues, as well as the features of its catalytic active site [62]. The available 3D structures for this nickel-dependent metalloenzyme show two conserved residues, arginine and a cysteine, both located on the substrate-covering loop (Figure 5). These residues are placed in the proximity of the Ni(II)-bound hydroxide anion (catalytic site) showing a good arrangement for covalent modification. The capacity of the cysteine residue to undergo chemical modifications was already demonstrated by Mazzei et al. that solved the crystal structure of Sp-URE inhibited by catechol (PDB ID 5G4H), along with other methyl substituted catechol derivatives (Figure 5a) [63,64]. These catechol derivatives were irreversible inhibitors that inactivate Sp-URE by avoiding the optimal conformation of the substrate-covering loop for catalysis (Figure 5c). The overlapping of the 3D structure of Sp-URE in complex with urea (PDB ID 6QDY, Figure 5a) [65], and PDB ID 5G4H (Figure 5b) [63] clearly shows a more open conformation of the loop (Figure 5c). Proteomic analysis of a covalently modified ribonuclease after treatment with glyoxal also revealed the conversion of the guanidinium group of arginine residues into five-membered heterocycles (imidazole-like) [66].

Therefore, the binding capability of MGO against Pa-URE was explored by docking to assess whether the predicted binding would be suitable for covalent modification of the conserved arginine residue R335. The interaction with Sp-URE was also compared using PDB ID 5G4H. A homology model from *P. aeruginosa* was created using the AlphaFold method [67,68]. The docking results revealed that MGO would bind close to the guanidinium group in residues R339 and R335 and in a suitable arrangement to trigger the chemical modification of these residues (Figure 5d,e). The simulation of the plausible modification of the arginine guanidium group as a 2-aminoimidazole derivative revealed that this modification would hinder, as happens with catechol, the appropriate folding of the substrate-covering loop over the metallic catalytic center as well as catalysis. In addition, more studies are needed to corroborate this hypothesis, the studies and background described herein suggest that both the intrinsic reactivity of MGO and its interaction with the urease enzyme would be adequate to alter the function of this important enzyme in relevant bacterial pathogens. Additional studies with the isolated enzyme would be necessary to corroborate this hypothesis. However, it is important to consider that this is preliminary information that would help further explore the MGO mechanism of action, specifically its interaction with the urease enzyme.

The results showed that MGO single and combined with LED/CMFs exhibited significant antimicrobial effects against *P. aeruginosa* and *C. albicans*. The treatments resulted in a lower impact on *S. aureus*. Cellular membrane studies revealed different results, suggesting that the antimicrobial activity is not related to the membrane. In addition to the antimicrobial activity of MGO and its combination with LED/CMFs, the current study indicates a possible effect against bacterial motility and enzymatic activity of *P. aeruginosa* which might affect the pathogenicity and invasion of the bacterium.

## 4. Materials and Methods

### 4.1. Experimental Plan

**Figure 6:**
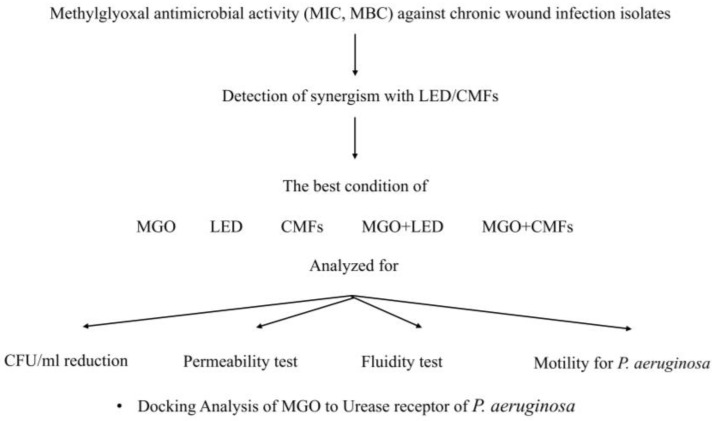
Experimental plan. MIC values of MGO were detected alone and in combination with LED and CMFs. The following experiments focused on the efficacy of the listed conditions on the CFU/mL reduction, permeability and fluidity of the microbial cell, the motility of *P. aeruginosa* strains, and docking analysis.

Figure 6 shows the experimental plan of this study.

### 4.2. Microbial Strains

Anonymized clinical *Staphylococcus aureus* PECHA 10 and *S. aureus* LMMV, *Pseudomonas aeruginosa* PECHA 4 and *P. aeruginosa* LMMV, and *Candida albicans* X3 strains were used for this study. The strains were isolated from chronic wounds of patients that gave their informed consent for the study that was approved by the Inter Institutional Ethic Committee of University “G. d’Annunzio” Chieti-Pescara, Chieti, Italy (ID n. richycnvw). *S. aureus* PECHA 10, and *P. aeruginosa* PECHA 4 were resistant strains described by Di Lodovico et al. [69] and Di Giulio et al. [4] *S. aureus* LMMV, *P. aeruginosa* LMMV and *C. albicans* X3 were characterized for their susceptibility to antibiotics/antifungals, ability to form biofilm [70,71], and virulence genes (*P. aeruginosa* LMMV for the *lasB* gene [72], *S. aureus* LMMV for *agr* genotype, *eta*, and *LukE-LukD*) [73,74,75].

The bacteria were cultured in Trypticase Soy Broth (TSB, Oxoid, Milan, Italy) and incubated at 37 °C for 24 h under aerobic conditions and then refreshed in TSB (1:10) for 2 h at 37 °C in an orbital shaker. Then, the broth cultures were standardized to optical density at 600 nm (OD_600_) = 0.12 (≈9.6 × 10^7^ CFU/mL). The broth culture of *Candida albicans*, grown on Sabouraud dextrose agar (SAB, Oxoid, Milan, Italy) was prepared in RPMI 1640 (Sigma-Aldrich, Milan, Italy) plus 2% glucose and standardized to OD_600_ = 0.15 (≈10^6^ CFU/mL).

### 4.3. Material and Devices

The material used in this study was Methylglyoxal and the devices were Light-Emitting Diodes and Complex Magnetic Fields, as indicated in Figure 6. Pyruvaldehyde solution (Methylglyoxal-MGO) 40 wt.% in H_2_O (Sigma-Aldrich, Milan, Italy) was used in the experiments. MGO application time was 22 min (which is similar to the application time of CMFs), while LED application was for 17 min.

The Light-Emitting Diode (LED) device (TL-06) characterized by a 630 nm ± 10 nm FHWM nm wavelength as the light source (Alpha strumenti, Melzo, Milan, Italy) was used to irradiate the samples. The handpiece constituted by 12 LEDS in a rectangular arrangement (3 × 4 LEDS) with 30 mW (site specific) irradiation covering a specific area. The application time was 17 min, as indicated by Di Lodovico et al. [76]. Light irradiation was performed by keeping the light sources stationary, in a perpendicular position, and at 5 cm from the samples.

As Complex Magnetic Fields Source, the C.M.F. machine, Next sx version (Medicina Fisica Integrata, M.F.I., Rome, Italy), that creates pulsed electromagnetic fields between 0.1 and 250 µT (emission intensity) and 1–250 Hz (frequency) was used. The magnets were made by winding 650 turns of 0.35 mm-wide enameled copper wire. The coil’s external dimensions are 110 mm, internal dimensions are 12 mm, and the coil’s thickness is 8 mm. Magnetic field lines were applied to the sample being treated at a 90° angle. Based on the results of a previous study [17], a specific program (ANTIBACTERIAL) in which multi-frequency magnetic fields of variable intensity, frequency, duration, and wave form, were administered to the samples with an application time of 22 min. The parameters of CMFs set in this study were as follows: frequency ranging from 6 to 70 Hz, magnetic induction of 6–95 µT, and exposure time of 22 min.

### 4.4. Antimicrobial Susceptibility Assay

The MGO antimicrobial action was evaluated by Minimum Inhibitory Concentration (MIC) and Minimum Bactericidal/Fungicidal Concentration (MBC/MFC) values. MIC was determined by the microdilution method, against standardized microbial suspensions of clinical strains used in this study (described above), as indicated by the Clinical and Laboratory Standards Institute [CLSI] [77] and according to Di Lodovico et al. [78]. For this test, 96 well microtiter plates (Nunc, Euro Clone SpA, Life Sciences-Division, Milan, Italy) were used. Two-fold dilutions of MGO stock solution ranging from 8192 to 8 µg/mL were performed in Trypticase Soy Broth (TSB, Oxoid, Milan, Italy) for bacteria and RPMI 1640 (Sigma-Aldrich, Milan, Italy) plus 2% glucose for *C. albicans* X3. A total of 100 µL of MGO and 100 μL of standardized microbial suspension were dispensed in each well of the 96 well microtiter plate and incubated for 24 h at 37 °C. The MIC values were measured by determining the lowest concentration of MGO able to inhibit the visible growth of the microorganisms. MBCs/MFCs were determined by subculturing 10 µL of suspensions from the MICs on Mueller Hinton agar (MHA, OXOID, Milan, Italy) for bacteria and on Sabouraud Dextrose agar (SAB, OXOID, Milan, Italy) for *C. albicans*.

### 4.5. Planktonic Optical Density

The effect of MGO (MIC values, 22 min), LED (17 min), and CMFs (22 min) on the growth of the microbial strains was determined by inoculating 100 µL of each standardized strain (following the previous standardization steps) into the well of 96 well microtiter plate in triplicate. For MGO, 100 µL of MGO solution (at each MIC values) and for LED and CMFs, 100 µL of the broth (TSB for bacteria and RPMI 1640 + 2% glucose for *C. albicans*) was added. After the end of application time, the OD_600_ values were detected using a microplate absorbance reader (BIO RAD, Milan, Italy) and compared to the control.

For the negative control, only medium (without the strains) and positive control (standardized broth cultures) were used to assess the test validity of the experimental protocol.

### 4.6. Synergism

The combined action of the studied materials and technologies against the microbial strains was evaluated. The synergisms between the MGO and LED/CMFs were evaluated using the checkerboard assay according to Di Fermo et al. [79]. For each treatment, MGO was added to the wells of each microbial strain followed by the application of (LED/CMFs) for the corresponding application time (17 min for LED, 22 min for CMFs). After the treatment, the microtiter plates were read with a microplate absorbance reader (BIO RAD, Milan, Italy). The synergistic effect of MGO combined with LED or CMFs was determined by the Fractional Inhibitory Concentration Index (FIC I) described by Odds as follows: synergism FIC I ≤ 0.5, antagonism FIC I ≥ 4.0, and additive FIC I > 0.5–4.0 [80].

### 4.7. CFU/mL Reduction

The broth cultures were standardized following the previously mentioned method. The bacteria were cultured in Trypticase Soy Broth (TSB, Oxoid, Milan, Italy) and incubated at 37 °C for 24 h under aerobic conditions and then refreshed in TSB (1:10) for 2 h at 37 °C in an orbital shaker. Then, the broth cultures were standardized to optical density at 600 nm (OD_600_) = 0.12 (≈9.6 × 10^7^ CFU/mL) followed by dilution 1:100 in TSB to obtain (≈10^5^ CFU/mL). The broth culture of *Candida albicans*, grown on Sabouraud dextrose agar (SAB, Oxoid, Milan, Italy) was prepared in RPMI 1640 (Sigma-Aldrich, Milan, Italy) plus 2% glucose and standardized to OD_600_ = 0.15 (≈10^6^ CFU/mL), treated according to the experimental plan (Figure 6) with LED (17 min), CMFs (22 min), and MGO at synergistic concentration, alone and combined with LED or CMFs.

For the evaluation of a viable and culturable cell count, the number of colony forming units per milliliter (CFU/mL) reduction was determined by spreading the treated samples on selective agar plates (Mannitol Salt Agar-MSA, Cetrimide-CET, Sabouraud-Agar, OXOID). Then, after incubation at 37 °C for 24 h, the CFU/mL was determined by calculating the average count of three agar plates for dilution.

### 4.8. Membrane Permeability

The effects of MGO (at synergistic concentration), LED, CMFs, MGO+LED, and MGO+CMFs on the membrane permeability of the studied strains (*S. aureus* PECHA 10, *S. aureus* LMMV, *P. aeruginosa* PECHA 4, *P. aeruginosa* LMMV, and *C. albicans* X3) was evaluated using propidium iodide (PI) as indicated by Di Lodovico et al. [17]. PI intercalates with bases of deoxyribonucleic acid (DNA), resulting in fluorescence. PI can enter the bacterial cell membrane only when it has been permeabilized through an agent and binds with DNA. This DNA-PI bound fluoresce has an excitation and emission of 544 nm and 620 nm, respectively.

The PI fluorescence was measured at an excitation and emission of 544 nm and 620 nm, through a fluorescence spectrophotometer (Agilent Technologies, Santa Clara, CA, USA).

### 4.9. Membrane Fluidity

The membrane fluidity of the studied strains in the presence and absence of MGO, alone and in combination with LED/CMFs, at the synergistic concentrations for each strain was determined by assessing the Laurdan generalized polarization (GPexc) as previously described [81]. The Laurdan emission spectra were obtained in a Varian Cary Eclipse fluorescence spectrofluorometer (Agilent Technologies, Santa Clara, CA, USA) at an excitation wavelength of 350 nm using emission wavelengths from 410 to 510 nm. The excitation GPexc was calculated using the following equation: GPexc = (I440 − I490)/(I440 + I490), where I440 and I490 are fluorescence intensities at 440 and 490 nm, respectively.

### 4.10. Swimming, Swarming, and Twitching Motility Assay

The capability of the tested treatments to interfere with the *P. aeruginosa* PECHA 4 and *P. aeruginosa* LMMV motility was established by swarming and swimming motility and for pilus retraction by twitching assay.

The swarming and the swimming motility tests were performed according to Abraham et al. [82] and the twitching motility assay according to Turnbull et al. [83]. Then, the bacterial halos were measured.

### 4.11. Building of the P. aeruginosa Model

The AlphaFold protein structure database homology modeling web server was used to model the three-dimensional structure of *P. aeruginosa* urease enzyme using the sequence UNIPROT Q9HUU5 [67,68].

### 4.12. Docking Studies

These studies were carried out using the GOLD program version 2021.3.0 [84]. The protein coordinates found in the crystal structure of the urease enzyme from *Sporosarcina pasteurii* (PDB ID 5G4H, 1.5 Å) were employed. Studies using the enzyme coordinates of the homology model of *P. aeruginosa* created were also conducted. The geometry of MGO was minimized using the AM1 Hamiltonian as implemented in the program Gaussian 09 [85] and used as an MOL2 file. The ligand was docked in 25 independent genetic algorithm (GA) runs, and for each of these a maximum number of 100,000 GA operations were performed on a single population of 50 individuals. Operator weights for crossover, mutation, and migration in the entry box were used as default parameters (95, 95, and 10, respectively), as well as the hydrogen bonding (4.0 Å) and van der Waals (2.5 Å) parameters. The position of residues R339 and R335 (for *S. pasteurii* and *P. aeruginosa*, respectively) was used to define the docking region and the radius of the selected spheric region was set to 6 Å. Except for the water molecules coordinated to the bimetallic catalytic site and hydroxide anion, all crystallographic water molecules and ligands were removed for docking. The “flip ring corners” flag was switched on, while all the other flags were off. The GOLD scoring function was used. The molecular graphics program PyMOL was employed for visualization and depicting ligand/protein structures. http://www.pymol.org/ (accessed on 27 January 2025) [86] (Figure S1).

### 4.13. Statistical Analysis

Statistical analysis was performed by using SPSS for Windows version 21 (IBM SPSS Inc., Chicago, IL, USA). Analysis of variance (ANOVA) and the Least Significant Difference (LSD) test were used to compare the parameters analyzed in the study for intra- and inter-group analysis. Data were obtained from at least three independent experiments performed in duplicate. *p* values less than 0.05 were considered significant.

## 5. Conclusions

Chronic wound infections represent a challenge that strongly underlines the search for new and eco-sustainable strategies. The results of this study showed antimicrobial activity of the mentioned single and combined treatments when applied to planktonic microorganisms coming from chronic wound infections with variations in the reduction percentage depending on the species. It was detected that the antimicrobial action was at the maximum with *C. albicans* and *P. aeruginosa*, and lower with *S. aureus* after the treatment. Also, the used treatments affected the cellular membrane determinants (permeability and fluidity) and the motility of *P. aeruginosa*. These single/combined treatments affected the microbial strains in terms of viable cell count, motility and cellular membrane permeability and fluidity. MGO docking analysis showed that the interaction between MGO and urease could alter the function of this enzyme. It is important to consider the safety of these technologies when applied following the studied protocol (intensity, frequency, and duration) and the eco-sustainability of the MGO and the devices. Therapy combination, which involves the simultaneous use of two or more antimicrobial agents could be integrated into wound dressings to test their direct biofilm inhibitory action. The in vitro studies can only provide insight to the in vivo situation, and these results justify further exploration to evaluate the effect of new technologies and their synergistic combination with MGO for the treatment of chronic infected wounds. The findings of this study suggest that combining MGO with LED or CMFs could have remarkable potential as a treatment for microbial chronic wound infections. Despite the limitations of this study, the obtained findings represent a valid and eco-sustainable approach to counteract chronic wound pathogens with an interesting impact on well-being. This strategy can be included in new wound bandages for correct wound management underling the sustainability of the proposal due to the use of non-antibiotic compounds and novel technologies with a low environmental impact.

## Figures and Tables

**Table 1:** Determination of Minimum Inhibitory Concentration (MIC, µg/mL) and Minimum Bactericidal Concentration (MBC/MFC, µg/mL) of MGO against the tested strains.

MGO	*S. aureus* PECHA 10	*S. aureus* LMMV	*P. aeruginosa* PECHA 4	*P. aeruginosa* LMMV	*C. albicans* X3
**MIC** (µg/mL)	64	128	256	256	4096
**MBC/MFC** (µg/mL)	64	128	256	256	4096

**Table 2:** Synergistic combinations of MGO (µg/mL) combined with LED (after 17 min of irradiation) or CMFs (after 22 min of irradiation) for all strains used in this study. * the used synergistic combination.

	Synergistic Combinations (MGO+LED) (µg/mL MGO)	FIC Index	Synergistic Combinations (MGO+CMFs) (µg/mL MGO)	FIC Index
*S. aureus* PECHA 10	16 * 32	0.250 0.500	16 * 32	0.250 0.500
*S. aureus* LMMV	8 * 16 32 64	0.062 0.125 0.250 0.500	2 * 4 8 16 32 64	0.015 0.031 0.062 0.125 0.250 0.500
*P. aeruginosa* PECHA 4	2 * 4 8 1 32 64 128	0.008 0.015 0.031 0.062 0.125 0.250 0.500	16 * 32 64 128	0.062 0.125 0.250 0.500
*P. aeruginosa* LMMV	16 * 32 64 128	0.062 0.125 0.250 0.500	16 * 32 64 128	0.062 0.125 0.250 0.500
*C. albicans* X3	16 * 32 64 128 256 512 1024 2048	0.004 0.008 0.015 0.031 0.062 0.125 0.250 0.500	4 * 8 16 32 64 128 256 512 1024	0.001 0.002 0.004 0.008 0.015 0.031 0.062 0.125 0.250

**Table 3:** GPexc values with MGO at synergistic concentrations (µg/mL) for each strain, LED, CMFs, best synergistic concentrations MGO+LED, MGO+CMFs; ND means not determined.

GPexc Values					
	*S. aureus* PECHA 10	*S. aureus* LMMV	*P. aeruginosa* PECHA 4	*P. aeruginosa* LMMV	*C. albicans* X3
**CTR**	0.12 ± 0.01	0.02 ± 0.003	0.03 ± 0.01	0.19 ± 0.07	−0.10 ± 0.005
**MGO 16**	0.06 ± 0.005	ND	0.12 ± 0.006	0.22 ± 0.001	0.11 ± 0.002
**MGO 8**	ND	0.17 ± 0.006	ND	ND	ND
**MGO 4**	ND	ND	ND	ND	−0.11 ± 0.004
**MGO 2**	ND	0.17 ± 0.0002	0.11 ± 0.01	ND	ND
**LED**	0.006 ± 0.006	0.15 ± 0.0004	0.11 ± 0.003	0.25 ± 0.003	−0.13 ± 0.004
**CMFs**	0.04 ± 0.0003	0.17 ± 0.0003	0.04 ± 0.005	0.25 ± 0.01	0.01 ± 0.01
**MGO + LED**	0.14 ± 0.006	0.02 ± 0.01	0.01 ± 0.001	0.04 ± 0.01	−0.14 ± 0.01
**MGO + CMFs**	0.11 ± 0.003	0.03 ± 0.01	0.05 ± 0.001	0.06 ± 0.002	0.06 ± 0.006

## Data Availability

The data that support the findings of this study are available from the corresponding author upon reasonable request.

## Supplementary Materials

The following supporting information can be downloaded at: https://www.mdpi.com/article/10.3390/antibiotics14040396/s1, Table S1. Antimicrobial susceptibility panel of the clinical strains used in this study; Table S2. *Staphylococcus aureus* LMMV, *Pseudomonas aeruginosa* LMMV and *C. albicans* X3 characterization for their capability to form biofilm and their main virulence factors; Figure S1. Amino acid sequence alignments for urease enzymes from distinct sources [MYCTU = *Mycobacterium tuberculosis*; SPOPA = *Sporosarcina pasteurii*; PSEAE = *Pseudomonas aeruginosa*; KLEAE = *Klebsiella aerogenes*; ECOLX = *Escherichia coli*]. Protein sequences were aligned using the CLUSTAL Omega multiple sequence alignment (version 1.2.4) (https://www.ebi.ac.uk/jdispatcher/msa/clustalo; accessed 10 April 2024). Relevant conserved residues are highlighted with a yellow shadow.

## Abbreviations

The following abbreviations are used in this manuscript:

AMR	Antimicrobial resistance
CFU/mL	Colony forming units per milliliter
CMFs	Complex Electromagnetic Fields
FIC I	Fractional Inhibitory Concentration Index
GPexc	Laurdan generalized polarization
LED	Light-Emitting Diodes
MGO	Methylglyoxal
MBC	Minimum Bactericidal Concentration
MFC	Minimum Fungicidal Concentration
MIC	Minimum Inhibitory Concentration
PDT	Photodynamic therapy
