# Supplementary material for: Methylglyoxal Alone or Combined with Light-Emitting Diodes/Complex Electromagnetic Fields Represent an Effective Response to Microbial Chronic Wound Infections
Source: Antibiotics (Basel). 2025 Apr 10;14(4):396. doi: 10.3390/antibiotics14040396 (PMC12024167; doi:10.3390/antibiotics14040396)
Supplement: Supplementary file 1 [file antibiotics-14-00396-s001.zip › antibiotics-3522683-supplementary.pdf]

Table S1. Antimicrobial susceptibility panel of the clinical strains used in this study.

	S. aureus **LMMV**	***P. aeruginosa*** **LMMV**	***Candida albicans*** **X3**
Levofloxacin	R	R	-
Netilmicin	S	I	-
Tetracycline	R	R	-
Erythromycin	I	R	-
Cefoxitin	S	R	-
Linezolid	S	R	-
Gentamicin	R	R	-
Ceftazidime	R	R	-
Cefuroxime	S	R	-
Ampicillin	R	R	-
Norfloxacin	I	S	-
Trim-Sulfamethoxazole	S	R	-
Nitrofurantoin	S	R	-
Vancomycin	R	R	-
Rifampicin	S	R	-
Teicoplanin	S	R	-
Ciprofloxacin	S	R	-
Clarithromycin	R	R	-
Amoxicillin	R	R	-
Amikacin	R	S	-
Amphotericin-B	-	-	S
Fluconazole	-	-	S
Flucytosine	-	-	S
Micafungin	-	-	S
Voriconazole	-	-	S

Table S2. *Staphylococcus aureus* LMMV, *Pseudomonas aeruginosa* LMMV and *C. albicans* X3 characterization for their capability to form biofilm and their main virulence factors.

	***S. aureus* LMMV**	***P. aeruginosa* LMMV**	***C. albicans X3***
Agr genotype	*Agr* 2	------	------
Exfoliative toxin *eta*	Negative	------	------
Leukotoxin *LukE-D*	Positive	------	------
Elastase *las B*	------	Positive	------
Biofilm producer	+++	+++	+++

```
sp|P9WFF1|URE1_MYCTU        -MARLSRERYAQLYGPTTGDRIRLADTNLLVEVTEDRCGGPGLAGDEAVFGGGKVLRESM
sp|P41020|URE1_SPOPA        --MKINRQQYAESYGPTVGDQVRLADTDLWIEVEKDTT----YGDEAVNFGGGKVLREGM
sp|Q9HUU5|URE1_PSEAE        --MKISRQAYADMFGPTVGDRVRLADTDLWIEVERDFT----VYGEEVKFGGGKVIRDGM
sp|P18314|URE1_KLEAE        -MSNISRQAYADMFGPTVGDKVRLADTELWIEVEDDLT----TYGEEVKFGGGKVIRDGM
tr|B1PCX0|B1PCX0_ECOLX      MMSNISRQAYADMFGPTTGDKIRLADTELWIEVEDDLT----TYGEEVKFGGGKVIRDGM
                               .:.*: **: :***.**::*****:* :**  *        .: . ******:*:.*

sp|P9WFF1|URE1_MYCTU        GQGRAS-RADGAPDTVITGAVIIDYWGIIKADIGIRDGRIVGIGKAGNPDIMTGVHRDLV
sp|P41020|URE1_SPOPA        GENGTYTRTENVLDLLLTNALILDYTGIYKADIGVKDGYIVGIGKGGNPDIMDGVTPNMI
sp|Q9HUU5|URE1_PSEAE        GQSQLG--AAQVVDTVITNALILDHWGVVKADVGLKDGRIQAIGKAGNPDIQPGVN--IA
sp|P18314|URE1_KLEAE        GQGQML--AADCVDLVLTNALIVDHWGIVKADIGVKDGRIFAIGKAGNPDIQPNVT--IP
tr|B1PCX0|B1PCX0_ECOLX      GQGQML--SAGCADLVLTNALIIDYWGIVKADIGVKDGRIFAIGKAGNPDIQPNVT--IP
                            *:.     :    * ::*.*:*:*: *: ***:*::** * .***.*****  .*   :

sp|P9WFF1|URE1_MYCTU        VGPSTEIISGNRRIVTAGTVDCHVHLICPQIIVEALAAGTTTIIGGGTGPAEGTKATTVT
sp|P41020|URE1_SPOPA        VGTATEVIAAEGKIVTAGGIDTHVHFINPDQVDVALANGITTLFGGGTGPAEGSKATTVT
sp|Q9HUU5|URE1_PSEAE        IGAGTEVIAGEGMILTAGGIDTHIHFICPQQIEEALMSGVTTMIGGGTGPAAGTNATTCT
sp|P18314|URE1_KLEAE        IGAATEVIAAEGKIVTAGGIDTHIHWICPQQAEEALVSGVTTMVGGGTGPAAGTHATTCT
tr|B1PCX0|B1PCX0_ECOLX      IGVSTEIIAAEGRIVTAGGVDTHIHWICPQQAEEALTSGTTTMIGGGTGPTAGSNATTCT
                            :* .**:*:.:  *:*** :* *:* * *:    **  * **:.******: *::*** *

sp|P9WFF1|URE1_MYCTU        PGEWHLARMLESLDGWPVNFALLGKGNTVNPDALWEQLRGGASGFKLHEDWGSTPAAIDT
sp|P41020|URE1_SPOPA        PGPWNIEKMLKSTEGLPINVGILGKGHGSSIAPIMEQIDAGAAGLKIHEDWGATPASIDR
sp|Q9HUU5|URE1_PSEAE        SGPWHMARMLQAADAFPMNIGFTGKGNASLPLPLEEQVLAGAIGLKLHEDWGSTPAAIDN
sp|P18314|URE1_KLEAE        PGPWYISRMLQAADSLPVNIGLLGKGNVSQPDALREQVAAGVIGLKIHEDWGATPAAIDC
tr|B1PCX0|B1PCX0_ECOLX      PGPWYIYQMLQAADSLPVNIGLLGKGNCSNPDALREQVAAGVIGLKIHEDWGATPAVINC
                             * * : :**:: :. *:*..: ***:      : **: .*. *:*:*****:*** *:

sp|P9WFF1|URE1_MYCTU        CLAVADVAGVQVALHSDTLNETGFVEDTIGAIAGRSIHAYHTEGAGGGHAPDIITVAAQP
sp|P41020|URE1_SPOPA        SLTVADEADVQVAIHSDTLNEAGFLEDTLRAINGRVIHSFHVEGAGGGHAPDIMAMAGHP
sp|Q9HUU5|URE1_PSEAE        CLEVAERHDIQVAIHTDTLNESGFVETTLGAFKGRTIHTYHTEGAGGGHAPDIIKACGFA
sp|P18314|URE1_KLEAE        ALTVADEMDIQVALHSDTLNESGFVEDTLAAIGGRTIHTFHTEGAGGGHAPDIITACAHP
tr|B1PCX0|B1PCX0_ECOLX      ALTVADEMDVQVALHSDTLNESGFVEDTLTAIGGRTIHTFHTEGAGGGHAPDIITACAHP
                            .* **:  .:***:*:*****:**:* *: *: ** **::*.***********:  ..

sp|P9WFF1|URE1_MYCTU        NVLPSSTNPTRPHTVNTLDEHLDMLMVCHHLNPRIPEDLAFAESRIRPSTIAAEDVLHDM
sp|P41020|URE1_SPOPA        NVLPSSTNPTRPFTVNTIDEHLDMLMVCHHLKQNIPEDVAFADSRIRPETIAAEDILHDL
sp|Q9HUU5|URE1_PSEAE        NVLPSSTNPTRPFTRNTIDEHLDMLMVCHHLDPAIAEDVAFAESRIRRETIAAEDILHDL
sp|P18314|URE1_KLEAE        NILPSSTNPTLPYTLNTIDEHLDMLMVCHHLDPDIAEDVAFAESRIRRETIAAEDVLHDL
tr|B1PCX0|B1PCX0_ECOLX      NILPSSTNPTLPYTVNTIDEHLDMLMVCHHLDPDIAEDVAFAESRIRQETIAAEDVLHDL
                            *:******** *.* **:*************.  * **:***:**** .******:***:

sp|P9WFF1|URE1_MYCTU        GAISMIGSDSQAMGRVGEVVLRTWQTAHVMKARRGALEGDPSGSQAADNNRVRRYIAKYT
sp|P41020|URE1_SPOPA        GIISMMSTDALAMGRAGEMVLRTWQTADKMKKQRGPLAEEKNG---SDNFRAKRYVSKYT
sp|Q9HUU5|URE1_PSEAE        GAFSMISSDSQAMGRVGEVITRTWQTADKMKRQRGRLDGDGAR---NDNFRARRYIAKYT
sp|P18314|URE1_KLEAE        GAFSLTSSDSQAMGRVGEVILRTWQVAHRMKVQRGALAEETGD---NDNFRVKRYIAKYT
tr|B1PCX0|B1PCX0_ECOLX      GAFSLTSSDSQAMGRVGEVVLRTWQVAHRMKVQRGPLPEESGD---NDNVRVKRYIAKYT
                            * :*: .:*: ****.**:: ****.*. ** :** *  :       ** *.:**::***

sp|P9WFF1|URE1_MYCTU        ICPAIAHGMDHLIGSVEVGKLADLVLWEPAFFGVRPHVVLKGGAIAWAAMGDANASIPTP
sp|P41020|URE1_SPOPA        INPAIAQGIAHEVGSIEEGKFADLVLWEPKFFGVKADRVIKGGIIAYAQIGDPSASIPTP
sp|Q9HUU5|URE1_PSEAE        INPAITHGISHEVGSVEAGKWADLVLWRPAFFGVKPSLILKGGAIAASLMGDINGSIPTP
sp|P18314|URE1_KLEAE        INPALTHGIAHEVGSIEVGKLADLVVWSPAFFGVKPATVIKGGMIAIAPMGDINASIPTP
tr|B1PCX0|B1PCX0_ECOLX      INPALTHGIAHEVGSIEVGKLADLVPWSPAFFGVKPATIVKGGMIAMAPMGDINGSIPTP
                            * **:::*: * :**:* ** **** * * ****:   ::*** ** : :** ..*****

sp|P9WFF1|URE1_MYCTU        QPVLPRPMFGAAAATAAATSVHFVAPQSIDARLADRLAVNRGLAPVADVRAVGKTDLPLN
sp|P41020|URE1_SPOPA        QPVMGRRMYGTVGDLIHDTNITFMSKSSIQQGVPAKLGLKRRIGTVKNCRNIGKKDMKWN
sp|Q9HUU5|URE1_PSEAE        QPVHYRPMFASYAGSRHATSLTFVSQAAFAAGVPQQLGLRKAIGVVSGCRGVQKTDLIHN
sp|P18314|URE1_KLEAE        QPVHYRPMFGALGSARHHCRLTFLSQAAAANGVAERLNLRSAIAVVKGCRTVQKADMVHN
tr|B1PCX0|B1PCX0_ECOLX      QPVHYRPMFAALGSARHRCRVTFLSQAAAANGVAEQLNLHSTTAVVKGCRTVQKADMRHN
                            ***  * *:.: .       : *::  :    :  :* :.   . * . * : * *:  *

sp|P9WFF1|URE1_MYCTU        DALPSIEVDPDTFTVRIDGQVWQPQPAAELPMTQRYFLF
sp|P41020|URE1_SPOPA        DVTTDIDINPETYEVKVDGEVLTCEPVKELPMAQRYFLF
sp|Q9HUU5|URE1_PSEAE        GYLPTIEVDAQNYQVRADGQLLWCEPADVLPMAQRYFLF
sp|P18314|URE1_KLEAE        SLQPNITVDAQTYEVRVDGELITSEPADVLPMAQRYFLF
tr|B1PCX0|B1PCX0_ECOLX      SLLPDITVDSQTYEVRINGELITSEPADILPMAQRYFLF
                            .    * :: :.: *: :*::   :*.  ***:******
```

Figure S1. Amino acid sequence alignments for urease enzymes from distinct sources [MYCTU = *Mycobacterium tuberculosis*; SPOPA = *Sporosarcina pasteurii*; PSEAE = *Pseudomonas aeruginosa*; KLEAE = *Klebsiella aerogenes*; ECOLX = *Escherichia coli*]. Protein sequences were aligned using the CLUSTAL Omega multiple sequence alignment (version 1.2.4) (https://www.ebi.ac.uk/jdispatcher/msa/clustalo; accessed April 10, 2024). Relevant conserved residues are highlighted with a yellow shadow.
